# Stabilizer codes for open quantum systems

**DOI:** 10.1038/s41598-023-37434-0

**Published:** 2023-06-29

**Authors:** Francisco Revson F. Pereira, Stefano Mancini, Giuliano G. La Guardia

**Affiliations:** 1 School of Science and Technology, University of Camerino, 62032 Camerino, Italy; 2 INFN, Sezione di Perugia, 06123 Perugia, Italy; 3 Department of Mathematics and Statistics, State University of Ponta Grossa, Ponta Grossa, PR 84030-900 Brazil; 4 Present Address: IQM, Nymphenburgerstr. 86, 80636 Munich, Germany

**Keywords:** Mathematics and computing, Physics

## Abstract

The Lindblad master equation describes the evolution of a large variety of open quantum systems. An important property of some open quantum systems is the existence of decoherence-free subspaces. A quantum state from a decoherence-free subspace will evolve unitarily. However, there is no procedural and optimal method for constructing a decoherence-free subspace. In this paper, we develop tools for constructing decoherence-free stabilizer codes for open quantum systems governed by the Lindblad master equation. This is done by pursuing an extension of the stabilizer formalism beyond the celebrated group structure of Pauli error operators. We then show how to utilize decoherence-free stabilizer codes in quantum metrology in order to attain the Heisenberg limit scaling with low computational complexity.

## Introduction

The second quantum revolution emerges from the possibility of designing and controlling quantum systems. The complexity of controlling quantum systems can be reduced by decreasing the noise due to system-environment interaction. This can be achieved by resorting to quantum error-correcting codes. Among them are the stabilizer codes^1^. Several works have extended the original construction method in order to incorporate Hilbert spaces and quantum systems with different structures^2–10^.

Stabilizer codes are often designed for a specific quantum channel, or anyway, their performance varies from channel to channel^11^. Having a dynamical evolution means dealing with time-varying Kraus operators, or equivalently, with time-varying quantum channels. Hence, in such a case, it might not be satisfactory to resort to the standard stabilizer code construction. In this paper, we consider an open quantum system described by the Lindblad master equation. This class of equations is the most general form for the generator of a quantum dynamical semigroup. We construct stabilizer codes able to eliminate the dissipator part of the Lindblad master equation, thus turning the evolution into unitary. As we show, this is possible since the stabilizer code corresponds to a decoherence-free subspace. A state from a decoherence-free subspace will evolve unitarily; i.e., the dissipator part of the Lindblad master equation will not contribute to the evolution of the state^12^. Although the stabilizer code constructed is a subspace of the corresponding decoherence-free subspace, an important advancement is made here. Applying the stabilizer code construction, we can derive a procedural and optimal method, in terms of computational complexity, for constructing the decoherence-free subspace that corresponds to the stabilizer code.

In doing so, we will also extend the stabilizer formalism to encompass the sum of error operators, besides their traditional composition. In other words, we will extend the formalism beyond the group structure of the error set, by considering a vector space structure for it. As a consequence, the standard dual structure of stabilizer codes^6–10,13–16^ will no longer be that of linear block codes in the general case, and the corresponding classical codes will be regarded as additive groups rather than vector spaces.

This paper is organized as follows. We initially present some concepts used to elaborate the results in this paper. A connection between stabilizer codes and decoherence-free subspaces is made. Next, we demonstrate the applicability of the stabilizer codes in the area of quantum metrology. A condition for probing a quantum system using stabilizer codes in order to obtain the Heisenberg limit scaling is stated and analyzed. Lastly, we suggest future lines of investigation from a coding theory perspective.

### Definitions

In this paper we deal with open quantum systems evolving by means of the Lindblad master equation. In order to address noise models that are not commonly considered in the literature of quantum error correction, we need to extended some concepts. Let the dynamics of the system’s density operator $\rho$ be given by^17^

1 $$\begin{aligned} \frac{\partial \rho }{\partial t} = -i[H_S,\rho ] + L_D(\rho ), \end{aligned}$$

where $L_D(\rho ) = \frac{1}{2}\sum _{l=1}^M \lambda _l ([J_l, \rho J_l^\dagger ] + [J_l\rho ,J_l^\dagger ])$ is the decoherence evolution originated from the system-reservoir coupling, with $M\le (2^N)^2 - 1$ where *N* is the number of qubits forming the system *S* (whose dimension is $2^N$) and $\{J_l\}_{l=1}^M$ are the Lindblad operators. We call this part of the evolution throughout the paper as the dissipator part. A decoherence-free subspace (DFS)^12^ ${\mathscr {H}}_\text {DFS}$ of ${\mathscr {H}}_S$ is such that all pure states $\rho (t)$ belonging to the set of density operators $D({\mathscr {H}}_\text {DFS})$ with support on ${\mathscr {H}}_\text {DFS}$ satisfy

2 $$\begin{aligned} \frac{d \text {Tr}\{\rho ^2(t)\}}{dt} = 0, \forall t\ge 0,\qquad \text { with }\qquad \text {Tr}\{\rho ^2(0)\}=1. \end{aligned}$$

On the other hand, a subspace ${\mathscr {H}}_\text {sDFS}$ is called *strong* decoherence-free subspace (sDFS) if for all pure $\rho (t)\in D({\mathscr {H}}_\text {sDFS})$ one has $L_D(\rho (t)) = 0,$ and $\rho ^2(t) = \rho (t), \forall t$. All conclusions drawn hereafter for decoherence-free subspace can be straightforwardly extended to *strong* decoherence-free subspace.

A stabilizer code ${\mathscr {Q}}$ is a subspace of a *N*-qubit system described by ${\mathbb {C}}^{2^N}$ stabilized by the elements of an abelian subgroup *S* of the error group $G_N$ over *N* qubits. The subgroup $C_{G_N}(S)$ of $G_N$, given by $C_{G_N}(S) = \{E\in G_N:E F = F E \text { for all }F\in S\},$ is called the centralizer of *S* in $G_N$. The center of $G_N$, denoted by $Z(G_N)$, is the subgroup $Z(G_N) = C_{G_N}(G_N)$. Let $S\le G_N$ be the stabilizer group of a stabilizer code ${\mathscr {Q}}$ of dimension greater than one. An error $E\in G_N$ is detectable by the stabilizer code ${\mathscr {Q}}$ if and only if *E* is an element of the set $\{sz:s \in S \text { and } z \in Z(G_N)\}$, or *E* does not belong to the centralizer $C_{G_N}(S)$^6^.

A set ${\mathscr {E}}$ of operators on ${\mathbb {C}}^2$ is denoted a *nice error basis* if it attains three conditions: (a) it contains the identity operator, (b) it is closed under the composition of operators, (c) $\text {Tr}\{A^\dagger B\} = 0$ for distinct elements $A,B\in {\mathscr {E}}$. In this paper, we consider the error basis ${\mathscr {E}} = \{{\mathbb {I}}, \sigma _x, \sigma _y, \sigma _z\},$ where ${\mathbb {I}}$ is the identity operator and $\sigma _i$, for $i=x,y,z$, are the Pauli matrices. The inner product of two distinct elements *A*, *B* in ${\mathscr {E}}$ is given by $\langle A,B\rangle = \text {Tr}\{A^\dagger B\}.$ Clearly, ${\mathscr {E}}$ is a nice error basis. Let ${\mathscr {E}}^N$ be the error basis constructed as *N*-fold tensor product of Pauli matrices described above. The error set, denoted by $G_N$, is the vector space over ${\mathbb {C}}$ consisting of elements in ${\mathscr {E}}^N$.

Let $\{\left| i\right\rangle \}_{i=1}^2$ be a basis of ${\mathbb {C}}^{2}$, and consider the $\left| i\right\rangle \left\langle j\right| \in {\mathscr {L}}({\mathbb {C}}^{2})$ (linear) operator over ${\mathbb {C}}^{2}$. The vectorization is a bijective linear map from ${\mathscr {L}}({\mathbb {C}}^{2})$ to ${\mathbb {C}}^{4}$ defined as^18^ $\text {vec}(\left| i\right\rangle \left\langle j\right| ):= \left| i\right\rangle \left| j\right\rangle .$ Such a map can be extended to any operator space. Several properties can be derived for matrix vectorization. Two operations that we use are composition and commutation of operators. For the first, we can exploit the relation

3 $$\begin{aligned} \text {vec}(ABC) = (A\otimes C^T)\text {vec}(B). \end{aligned}$$

In particular, we have $\text {vec}(AB) = (A\otimes {\mathbb {I}})\text {vec}(B)$. The commutator can be easily obtained from the above relation and by the linearity of the vectorization. In particular, we have

4 $$\begin{aligned} \text {vec}([A,B]) = (A\otimes {\mathbb {I}} - {\mathbb {I}}\otimes A^T)\text {vec}(B). \end{aligned}$$

## Results

The error set in the standard stabilizer formalism is given by a set of operators whose elements obey the usual composition of operators. In this paper, operators can also be summed, thus leading to a vector space structure for the error set. Notice, however, that to formulate the stabilizer code construction in both approaches (the standard one and the one used in this paper) one only needs to utilize the composition of operators, besides the commutativity of its elements.

Suppose the evolution of a state $\rho (t)$ is given by the Lindblad master equation with dissipator part described by operators from the set ${\mathscr {J}} = \{J_l:l=1,\dots , M\}$. Assume that there exists a DFS ${\mathscr {H}}_\text {DFS} = \text {span}\{\left| \psi _i\right\rangle \}_{i=1,\ldots ,K}$ and that $J_l\left| \psi _k\right\rangle = c_l\left| \psi _k\right\rangle$, for all $l=1,\ldots ,M$ and $k = 1,\ldots , K$. We can construct the following stabilizer set ${\mathscr {S}}_\text {DFS} := \langle S_1, \ldots , S_{M}:S_l = c_l^{-1} J_l, \text { for }l=1,\ldots ,M,\text { where }J_l\in {\mathscr {J}}\rangle .$ Suppose there exists a nontrivial maximal joint $+1$-eigenspace ${\mathscr {Q}}$ of the abelian group of ${\mathscr {S}}_\text {DFS}$. Then, define $H_{ev} = H_S + \frac{i}{2}\sum _{l = 1}^M \lambda _l(c_l^* J_l - c_l J_l^\dagger )$. If it belongs to $C_{G_N}({\mathscr {S}}_\text {DFS})$, then ${\mathscr {Q}}$ is a stabilizer code and a decoherence-free subspace (see Subsection Stabilizer Codes and Decoherence-Free Subspaces of Methods). We call ${\mathscr {Q}}$ a decoherence-free stabilizer code.

The connection between decoherence-free subspaces and stabilizer codes is expanded in the following two subsections. Firstly, errors with a particular structure are considered. This structure simplifies the stabilizer formalism and the connection between stabilizers and classical codes. Afterwards, the restriction is relaxed and generalized errors are considered.

### Decoherence-free stabilizer codes for tensor-product noise

Let *N* be a positive integer, and $E_1,E_2$ be two errors written as

5 $$\begin{aligned} E_1= & {} \bigotimes _{j=1}^N \Big {(}a_{0j}{\mathbb {I}}_{j} + a_{1j}\sigma _{xj} + a_{2j}\sigma _{yj} + a_{3j}\sigma _{zj}\Big {)}, \end{aligned}$$

6 $$\begin{aligned} E_2= & {} \bigotimes _{j=1}^N \Big {(}b_{0j}{\mathbb {I}}_{j} + b_{1j}\sigma _{xj} + b_{2j}\sigma _{yj} + b_{3j}\sigma _{zj}\Big {)}. \end{aligned}$$

Let ${\tilde{G}}_N\subseteq {G}_N$ be the set containing elements of the form $E_1,E_2$ above. Then we define the map

7 $$\begin{aligned} \begin{aligned} \zeta :{\tilde{G}}_N&\rightarrow {\mathbb {C}}^{4N},\\ \bigotimes _{j=1}^N \Big {(}a_{0j}{\mathbb {I}}_{j} + a_{1j}\sigma _{xj} + a_{2j}\sigma _{yj} + a_{3j}\sigma _{zj}\Big {)}&\mapsto (a_{01}, \ldots , a_{0N}, a_{11}, \ldots , a_{1N}, a_{21}, \ldots , a_{2N}, a_{31}, \ldots a_{3N}) \end{aligned} \end{aligned}$$

by means of the operation

8 $$\begin{aligned} \zeta (E_1 E_2) = \left( \begin{array}{llllllllllll} c_{01}, &{} \ldots , &{} c_{0N}, &{} c_{11}, &{} \ldots , &{} c_{1N}, &{} c_{21}, &{} \ldots , &{} c_{2N}, &{} c_{31}, &{} \ldots , &{} c_{3N}\\ \end{array}\right) , \end{aligned}$$

where

9a $$\begin{aligned} c_{0j}= & {} a_{0j} b_{0j} + a_{1j} b_{1j} + a_{2j} b_{2j} + a_{3j} b_{3j}, \end{aligned}$$

9b $$\begin{aligned} c_{1j}= & {} (a_{1j} b_{0j} + a_{0j} b_{1j}) + i(a_{2j} b_{3j} - a_{3j} b_{2j}), \end{aligned}$$

9c $$\begin{aligned} c_{2j}= & {} (a_{2j} b_{0j} + a_{0j} b_{2j}) + i(a_{3j} b_{1j} - a_{1j} b_{3j}), \end{aligned}$$

9d $$\begin{aligned} c_{3j}= & {} (a_{3j} b_{0j} + a_{0j} b_{3j}) + i(a_{1j} b_{2j} - a_{2j} b_{1j}), \end{aligned}$$

for $j=1,\ldots ,N$. On the other hand, let ${\varvec{v}}_1,{\varvec{v}}_2\in {\mathbb {C}}^{4N}$ be two vectors given, respectively, by

10 $$\begin{aligned} {\varvec{v}}_1= & {} \left( \begin{array}{lllllllllllll} a_{01},&\ldots ,&a_{0N},&a_{11},&\ldots ,&a_{1N},&a_{21},&\ldots ,&a_{2N},&a_{31},&\ldots ,&a_{3N} \end{array}\right) , \end{aligned}$$

11 $$\begin{aligned} {\varvec{v}}_2= & {} \left( \begin{array}{lllllllllllll} b_{01},&\ldots ,&b_{0N},&b_{11},&\ldots ,&b_{1N},&b_{21},&\ldots ,&b_{2N},&b_{31},&\ldots ,&b_{3N} \end{array}\right) . \end{aligned}$$

Define the binary operation $+_{\zeta }$ as

12 $$\begin{aligned} {\varvec{v}}_1 +_{\zeta } {\varvec{v}}_2:= \left( \begin{array}{llllllllllll} c_{01},&\ldots ,&c_{0N},&c_{11},&\ldots ,&c_{1N},&c_{21},&\ldots ,&c_{2N},&c_{31},&\ldots ,&c_{3N} \end{array}\right) , \end{aligned}$$

where $c_{0j}$, $c_{1j}$, $c_{2j}$, and $c_{3j}$, for $j=1,\ldots ,N$, are given in Eqs. (9a-9d).

Let *N* be a positive integer and ${\mathscr {V}} = \{{\varvec{v}}\in {\mathbb {C}}^{4N}|{\varvec{v}} = ({\varvec{x}}_0, {\varvec{x}}_1, {\varvec{x}}_2, {\varvec{x}}_3)\text { where }{\varvec{x}}_0 = (1, 1,\ldots ,1)\in {\mathbb {C}}^N\text { and }{\varvec{x}}_1,{\varvec{x}}_2,{\varvec{x}}_3\in {\mathbb {C}}^N\}$ be a group under $+_\zeta$. Then the maps

13 $$\begin{aligned}{} & {} \langle \cdot , \cdot \rangle _{\zeta _{(1,j)}}:{\mathbb {C}}^{4N}\times {\mathbb {C}}^{4N}\rightarrow {\mathbb {C}}\nonumber \\{} & {} \quad ({\varvec{v}}_A,{\varvec{v}}_B)\mapsto \langle {\varvec{v}}_A,{\varvec{v}}_B\rangle _{\zeta _{(1,j)}} = (a_{2j} b_{3j} - a_{3j} b_{2j}), \end{aligned}$$

14 $$\begin{aligned}{} & {} \langle \cdot , \cdot \rangle _{\zeta _{(2,j)}}:{\mathbb {C}}^{4N}\times {\mathbb {C}}^{4N}\rightarrow {\mathbb {C}}\nonumber \\{} & {} \quad ({\varvec{v}}_A,{\varvec{v}}_B)\mapsto \langle {\varvec{v}}_A,{\varvec{v}}_B\rangle _{\zeta _{(2,j)}} = (a_{3j} b_{1j} - a_{1j} b_{3j}), \end{aligned}$$

15 $$\begin{aligned}{} & {} \langle \cdot , \cdot \rangle _{\zeta _{(3,j)}}:{\mathbb {C}}^{4N}\times {\mathbb {C}}^{4N}\rightarrow {\mathbb {C}}\nonumber \\{} & {} \quad ({\varvec{v}}_A,{\varvec{v}}_B)\mapsto \langle {\varvec{v}}_A,{\varvec{v}}_B\rangle _{\zeta _{(3,j)}} = (a_{1j} b_{2j} - a_{2j} b_{1j}), \end{aligned}$$

are symplectic forms over ${\mathscr {V}}$, where ${\varvec{v}}_A = ({\varvec{x}}_0, {\varvec{a}}_1, {\varvec{a}}_2, {\varvec{a}}_3)$, ${\varvec{v}}_B = ({\varvec{x}}_0, {\varvec{b}}_1, {\varvec{b}}_2, {\varvec{b}}_3)$, and $j=1,\ldots ,N$ (see Subsection Symplectic form and Additive Codes of Methods).

Now, we have the tools to define the symplectic dual of an $+_\zeta$-additive code. Let *N* be a positive integer and $C = \{{\varvec{c}}\in {\mathbb {C}}^{4N}|{\varvec{c}} = ({\varvec{c}}_0, {\varvec{c}}_1, {\varvec{c}}_2, {\varvec{c}}_3),\text { where }{\varvec{c}}_0 = (1, 1,\ldots ,1)\in {\mathbb {C}}^N\text { and }{\varvec{c}}_1,{\varvec{c}}_2,{\varvec{c}}_3\in {\mathbb {C}}^N\}$ be an $+_\zeta$-additive code. The symplectic dual of *C* is given by

16 $$\begin{aligned} C^{\perp _\zeta }:= \{{\varvec{c}}\in {\mathbb {C}}^{4N}:\langle {\varvec{c}}, {\varvec{d}}\rangle _{\zeta _{(l,j)}} = 0, \text { for all }{\varvec{d}}\in C, l=1,2,3, \text { and }j=1,\ldots ,N\}. \end{aligned}$$

Similarly to previous works on stabilizer codes, we are going to derive a connection between stabilizer codes and classical error-correcting codes. This approach enables us to derive algebraic conditions for the construction and existence of decoherence-free stabilizer codes. We can use it to show nonexistence of decoherence-free stabilizer codes with some specific parameters.

#### Theorem 1

Let ${\mathscr {V}}_{{\mathscr {S}}_\text {DFS}} = \zeta ({\mathscr {S}}_\text {DFS})$ be a basis of the $+_\zeta$-additive code of the form $C = \{{\varvec{c}}\in {\mathbb {C}}^{4N}|{\varvec{c}} = ({\varvec{c}}_0, {\varvec{c}}_1, {\varvec{c}}_2, {\varvec{c}}_3)\text { where }{\varvec{c}}_0 = (1, 1,\ldots ,1)\in {\mathbb {C}}^N\text { and }{\varvec{c}}_1,{\varvec{c}}_2,{\varvec{c}}_3\in {\mathbb {C}}^N\}$. Then, a decoherence-free stabilizer code ${\mathscr {Q}}$ exists if there exists an $+_\zeta$-additive code *C* over ${\mathbb {C}}$ generated by ${\mathscr {V}}_{{\mathscr {S}}_\text {DFS}}$ such that $C\le C^{\perp _\zeta }$ and $\zeta (H_{ev})\in C^{\perp _\zeta }.$

For further explanation, see “Decoherence-Free Stabilizer Codes for Tensor-Product Noise” and “Symplectic form and Additive Codes of Methods” subsections.

As can be noticed in Theorem 1, one needs that $C\le C^{\perp _\zeta }$ for constructing stabilizer codes from classical error-correcting codes. Such an expression is required to guarantee that ${\mathscr {Q}}\le C_{G_N}({\mathscr {Q}})$^6^.

### Decoherence-free stabilizer codes for general noise

Let ${\mathscr {S}}$ be a stabilizer group with operators satisfying the structure of the standard stabilizer formalism. Assume that ${\mathscr {C}}$ is the additive group constructed using the standard stabilizer formalism and ${\mathscr {C}}_\text {vec} = \text {vec}({\mathscr {S}})$, where the composition of operators in ${\mathscr {S}}$ corresponds to the respective operation of the additive group. Then ${\mathscr {C}} \equiv {\mathscr {C}}_\text {vec}$ (see subsection Decoherence-Free Stabilizer Codes for General Noise of Methods).

As explained in the previous subsection, we need to have a symplectic form in order to construct the additive code related to the stabilizer code and its centralizer. We can use Eq. (4) to construct the symplectic form used in this subsection. Let $A,B\in {\mathscr {L}}({\mathbb {C}}^{2N})$ be linear operators. We define the map

17 $$\begin{aligned} \begin{aligned} \langle \cdot , \cdot \rangle _{\text {vec}}:{\mathbb {C}}^{2N}\times {\mathbb {C}}^{2N}&\rightarrow {\mathbb {C}}\\ (\text {vec}(A),\text {vec}(B))&\mapsto \langle \text {vec}(A),\text {vec}(B)\rangle _\text {vec} = \sum _{i=1}^{2N}[(A\otimes {\mathbb {I}} - {\mathbb {I}}\otimes A^T)\text {vec}(B)]_i. \end{aligned} \end{aligned}$$

The above map turns out to be a symplectic form over ${\mathbb {C}}$ (see Subsection Symplectic form and Additive Codes of Methods).

Since $\langle \cdot , \cdot \rangle _{\text {vec}}$ gives a symplectic form, we can define the dual code of an additive code. Furthermore, we can extend the stabilizer formulation presented in the previous subsection to a larger set of errors. Let *C* be an $+_\text {vec}$-additive code. The symplectic dual of *C* is given by

18 $$\begin{aligned} C^{\perp _\text {vec}}:= \{{\varvec{c}}\in {\mathbb {C}}^{2N}:\langle {\varvec{c}}, {\varvec{d}}\rangle _\text {vec} = 0, \text { for all }{\varvec{d}}\in C\}. \end{aligned}$$

#### Theorem 2

Let ${\mathscr {V}}_{{\mathscr {S}}_\text {DFS}} = \text {vec}({\mathscr {S}}_\text {DFS})$ be a basis of the $+_\text {vec}$-additive code *C*. Then, a decoherence-free stabilizer code ${\mathscr {Q}}$ exists if there exists an $+_\text {vec}$-additive code *C* over ${\mathbb {C}}$ generated by ${\mathscr {V}}_{{\mathscr {S}}_\text {DFS}}$ such that $C\le C^{\perp _\text {vec}}$ and $\text {vec}(H_{ev})\in C^{\perp _\text {vec}}$.

Notice that Theorem 2 extends the result presented in Theorem 1 for general noise. For further explanation and discussions, see “Decoherence-Free Stabilizer Codes for General Noise” subsection.

## Discussion

### Application to Parameter Estimation

Suppose we have a unitary evolution given by $U = \exp (-i H_S)$, where $H_S = \eta H$ is the system Hamiltonian, $\eta$ is a parameter to be estimated, and *H* is the generator of *U*. One of the goals of quantum metrology is to reduce the error obtained in estimating $\eta$ when compared to classical strategies. To attain this goal, we use *N* identical and independent probes, measure them in the channel output, and average the results. Such scheme has the estimation precision lower bounded by^19,20^

19 $$\begin{aligned} \Delta \eta \Delta h\ge \frac{1}{2}, \end{aligned}$$

where $\Delta A$ is the standard deviation of the random variable *A*, and $h = \sum _{j=1}^N H_j$, $H_j$ acting on the *j*-th probe, stands for the generator of the unitary evolution $U^{\otimes N}$. It is shown in Ref.^21^ that there exists a probing state and a measurement strategy such that

20 $$\begin{aligned} \Delta \eta \ge \frac{1}{N(\lambda _\text {Max} - \lambda _\text {Min})}, \end{aligned}$$

where $\lambda _\text {Max}$ and $\lambda _\text {Min}$ are, respectively, the maximum and minimum eigenvalues of *h*. This is accomplished with the use of general probe states, which may be entangled states, and local or joint measurements after the unitary evolution $U^{\otimes N}$. When the standard deviation (20) scales like 1/*N*, we say that it attains the Heisenberg limit (HL) scaling.

A crucial assumption used in the above methodology to attain the HL is that evolution is unitary. For Markovian noise, one alternative approach is to use a quantum error-correcting code to achieve the HL under the assumption that the system Hamiltonian is not in the spanned space generated by the Lindblad operators^22–26^. Refs^22,23^ show that lower bounds can be constructed from a simple algebraic condition involving solely the operators appearing in the quantum master equation. A preliminary protocol considering the requirements that quantum error-correcting codes must satisfy to achieve HL is also described in Ref.^23^. This proposal has been further extended for general adaptive multi-parameter estimation schemes in the presence of Markovian noise^26^. Lastly, Ref.^25^ gives a semidefinite program for finding optimal ancilla-free sensing codes.

The proposed protocol of this paper is described as follows. The first part is the construction of the stabilizer code from the open quantum system evolution. Let $\rho _{\text {Max-Min}}$ be the equally weighted superposition of the eigenvectors relative to the maximum and minimum eigenvalues of $\sum _{i=1}^N \mathbbm {1}^{\otimes i-1}_S\otimes H_S\otimes \mathbbm {1}^{\otimes N-i}_S$. Next, we see if the stabilizer code contains the state $\rho _{\text {Max-Min}}$. If so, then we use it to probe the quantum system. As shown in the previous section, we are going to have a unitary evolution described by $H_S$. Therefore, using the optimal measurement described in Ref.^21^ over the channel outputs, one obtains the HL scaling. We give a formal description of our protocol below.

The present idea differs from the literature on the use of quantum codes to attain the HL^22–24,26^ in terms of computational complexity. Here, we do not need to implement a decoding process, which is the case of Refs^22–25^. However, this decoder-free approach is not novel in the literature, e.g. Ref.^26^ proposes a semidefinite program design to identify the optimal quantum error-correcting protocol, without the necessity for a decoding algorithm, to achieve the best estimation precision in cases where the Heisenberg scaling is attainable. The quantum state will not change by the environmental noise since it belongs to the DFS. Therefore, there is no error to be detected or corrected. Removing the decoder from the picture, we have a reduced number of operations to be implemented and a faster probing strategy.

Consider a quantum system with evolution given by the Lindblad master equation with Lindblad operators $\{J_l\}$. Let ${\mathscr {S}}$ be a stabilizer set constructed from the Lindblad operators. Let $\left| \psi _{\text {max}}\right\rangle$ and $\left| \psi _{\text {min}}\right\rangle$ be eigenvectors of the system Hamiltonian $H_S$ with maximum and minimum eigenvalues, respectively. Then, Heisenberg limit scaling is achievable if

21 $$\begin{aligned} \left| \psi ^{(N)}\right\rangle = \frac{1}{\sqrt{2}}(\left| \psi _{\text {max}}\right\rangle ^{\otimes N} + \left| \psi _{\text {min}}\right\rangle ^{\otimes N}) \end{aligned}$$

belongs to the stabilizer code for any $N>N^*$, where $N^*\in {\mathbb {N}}$. This is a clear application of the formulation constructed in the previous section and the methodology of achieving the Heisenberg scaling from Ref.^21^. In fact, since $\left| \psi ^{(N)}\right\rangle$ belongs to the stabilizer code, then it also belongs to the DFS, hence its evolution is unitary and the technique of Ref.^21^ can be applied.

We use the above formulation in the example below to show the achievability of the HL scaling. The proposed protocol relies on $\rho _{\text {Max-Min}}$ as a codeword of the DFS stabilizer code. The existence of a DFS stabilizer code is equivalent to the commutativity between the Lindblad operators and the system Hamiltonian. This is satisfied whenever we have environments acting locally on each subsystem. Therefore, we expect that the proposed protocol can be applied to most of the relevant physical systems.

#### Example

Consider a quantum system with the dynamics governed by

22 $$\begin{aligned} \frac{\partial \rho }{\partial t} = -i[H_S,\rho ] + \frac{\gamma }{2}(2J\rho J^\dagger - J^\dagger J\rho - \rho J^\dagger J), \end{aligned}$$

where

23 $$\begin{aligned} J = \frac{s+c}{2}({\mathbb {I}}\otimes {\mathbb {I}} + \sigma _{z}\otimes \sigma _z), \end{aligned}$$

and

24 $$\begin{aligned} H_S = \frac{\gamma (s+c)^2}{4}(\sigma _{x}\otimes \sigma _x), \end{aligned}$$

with $s = \sinh (r)$, $c=\cosh (r)$, and *r* is the (real) squeezing parameter. The stabilizer set constructed from the dissipator part is given by ${\mathscr {S}} = \langle ({\mathbb {I}}\otimes {\mathbb {I}} + \sigma _{z}\otimes \sigma _z)^i:i=0,1\rangle$. Consider an eigenvector with maximum eigenvalue and an eigenvector with minimum eigenvalue of the operator $H_S$. Such a pair is

25 $$\begin{aligned} \left| \psi _{\text {Max}}\right\rangle = \frac{1}{\sqrt{2}}(\left| 00\right\rangle + \left| 11\right\rangle ) \qquad \text {and} \qquad \left| \psi _{\text {Min}}\right\rangle = \frac{1}{\sqrt{2}}(\left| 00\right\rangle - \left| 11\right\rangle ). \end{aligned}$$

Suppose we are going to probe the system *N* times with the state

26 $$\begin{aligned} \rho _{\text {Max-Min}} = \left| \psi ^{(N)}\right\rangle \left\langle \psi ^{(N)}\right| = \frac{1}{2}\Big {(}\left| \psi _{\text {Max}}\right\rangle ^{\otimes N} + \left| \psi _{\text {Min}}\right\rangle ^{\otimes N}\Big {)}\Big {(}\left\langle \psi _{\text {Max}}\right| ^{\otimes N} + \left\langle \psi _{\text {Min}}\right| ^{\otimes N}\Big {)}. \end{aligned}$$

It is possible to see that $\left| \psi ^{(N)}\right\rangle$ is a codeword of the stabilizer code ${\mathscr {Q}}$, since $S\left| \psi _{\text {Max}}\right\rangle = \left| \psi _{\text {Max}}\right\rangle$ and $S\left| \psi _{\text {Min}}\right\rangle = \left| \psi _{\text {Min}}\right\rangle$, for all $S\in {\mathscr {S}}$. Now, the achievability of the HL scaling can be seen in two ways. Firstly from the above discussion, where state membership in the stabilizer code is verified in the quantum or classical realms using the tools presented previously in this paper. Secondly from Eq. (22), where we have that the dissipator part does not contribute to the evolution since

27 $$\begin{aligned} 2J\rho _{\text {Max-Min}} J^\dagger - J^\dagger J\rho _{\text {Max-Min}} - \rho _{\text {Max-Min}} J^\dagger J = 2\rho _{\text {Max-Min}} - \rho _{\text {Max-Min}} - \rho _{\text {Max-Min}} = 0. \end{aligned}$$

### Concluding remarks

In this work we have constructed stabilizer codes for open quantum systems governed by the Lindblad master equation. To achieve this goal, we had to go beyond the tools that exist for stabilizer codes in the literature. As an important step, we have extended the formulation of stabilizer codes under the influence of errors forming a group to those forming a vector space. Using stabilizer codes as tools, we were able to determine conditions under which decoherence-free subspaces exist.

Observe that we have not been the first to identify a connection between stabilizer codes and decoherence-free subspaces. However, differently from previous works^27^, we give a direct algebraic relation between the Lindblad operators, DFSs, and stabilizer codes. It is shown in Ref.^27^ that DFSs are a specific class of quantum error correcting codes, but no constructive method to derive the stabilizer set from the Lindblad operators was shown. Furthermore, as was shown in previous sections, we extended the stabilizer description to classical error-correcting codes defined over the complex number field. More precisely, the standard theory of quantum error-correcting codes contains quantum codes derived from classical codes, i.e., linear codes defined over finite fields. In this new context, we consider classical codes defined over ${\mathbb {C}}$, the complex field which has characteristic zero, and this fact modifies completely the techniques to be applied in the constructions of our results. To the best of the author’s knowledge, this is the first work presenting such a formulation. In particular, there are DFS that have a stabilizer code as a subspace. This inclusion may or may not be proper. However, dealing with stabilizer codes can produce results that we could not obtain otherwise. In fact, one can find encoding methods for stabilizer codes that are procedural and optimum algorithms for creating the corresponding code space. Additionally, set membership can be optimally implemented by decoding methods. Later in the paper we constructed an algorithm for quantum metrology that uses set membership as one of the important steps. Therefore, dealing with decoherence-free stabilizer code instead of the whole decoherence-free subspace is computationally relevant for several applications.

It is worth noting that the methodology taken to develop the symplectic dual and decoherence-free stabilizer codes can be tailored to general noise. Suppose we wish to extend the formulation of to operators of the form

28 $$\begin{aligned} E = \sum _{l=1}^L \bigotimes _{j=1}^N (a_{0j}^l{\mathbb {I}}_{j}+ a_{1j}^l\sigma _{xj} + a_{2j}^l\sigma _{yj} + a_{3j}^l\sigma _{zj}), \end{aligned}$$

where *L* is the number of terms in the sum describing the operator *E*, and *N* is the number of physical systems. A naive approach would be to map operators to matrices where, for a fixed *l*, the elements $a_{ij}^l$, for $i=0,1,2,3$ and $j=1,\ldots ,N$, correspond to the *l*-th row of the respective matrix. There are some problems with this strategy. First of all, one should impose an ordering over the terms in the sum going from $l=1,\ldots ,L$ as a way to make a uniquely correspondence between each term in the sum and a row in the matrix. Secondly, the composition of errors could result in a sum of matrices giving a matrix with more rows than the original matrices that are being summed; e.g., suppose we have $E_1$ with $L_1$ terms in the sum and $E_2$ with $L_2$ terms in the sum, then $E_1\circ E_2$ can produce up to $L_1\times L_2$ terms. This can be solved since there is a maximum $L'$ of terms with which any operator can be described. Third, and more importantly, the above representation is not unique. To see this, consider the operator

29 $$\begin{aligned} A = ({\mathbb {I}} + \sigma _x + \sigma _z)\otimes \sigma _y + \sigma _z\otimes \sigma _x, \end{aligned}$$

which can also be written as

30 $$\begin{aligned} A = ({\mathbb {I}}+\sigma _x)\otimes \sigma _y + \sigma _z\otimes (\sigma _x + \sigma _y). \end{aligned}$$

The issue of uniqueness in representing an operator and, consequently, its matrix representation may be solved by introducing equivalence classes over matrix spaces similar to the equivalence classes utilized in the definition of tensor product of vector spaces^28^. Even though this problem may be solved, the formulation seems not straightforward. Therefore, we have used matrix vectorization to avoid all these complications.

This paper suggests future lines of investigation from a coding theory perspective. Firstly, constructing code parameter bounds by connecting physical constraints over Lindblad operators to the stabilizer code parameters. A quantification of goodness for decoherence-free subspaces can be obtained from this topic. One could also show the nonexistence of decoherence-free subspaces, which could lead to a more effective approach to investigating open quantum systems. Secondly, identifying decoherence-free subspaces as stabilizer codes generates the possibility to classify some evolutions of open quantum systems. One approach is connecting some evolutions to families of classical codes. Lastly, because of the novel approach presented, we expect quantum evolutions with decoherence-free stabilizer codes leading to classical codes that have not been discovered yet.

As an application of our formulation, we presented a novel algebraic method for attaining the Heisenberg limit scaling is using of stabilizer codes. Explanations of tools and codes created in the paper are illustrated through an example. The algebraic approach developed to attain the Heisenberg limit scaling paves the way to attack this quantum metrology problem by reservoir engineering. Finally, we would like to point out that our formalism applies also to Lindbladian operators coming from microscopic (Hamiltonian) dynamics, although the realizability of decoherence-free stabilizer codes will of course then depend on such dynamics, and will, eventually, be related to the existence of dark states.

## Methods

### Stabilizer codes and decoherence-free subspaces

Now, we are going to describe in detail the error basis and error vector space used throughout the paper. A set ${\mathscr {E}}$ of operators on ${\mathbb {C}}^2$ is denoted a *nice error basis* if it attains three conditions: (a) it contains the identity operator, (b) it is closed under the composition of operators, (c) $\text {Tr}\{A^\dagger B\} = 0$ for distinct elements $A,B\in {\mathscr {E}}$. In this paper, we consider the error basis

31 $$\begin{aligned} {\mathscr {E}} = \{{\mathbb {I}}, \sigma _x, \sigma _y, \sigma _z\}, \end{aligned}$$

where ${\mathbb {I}}$ is the identity operator and $\sigma _i$, for $i=x,y,z$, are the Pauli matrices. The inner product of two distinct elements *A*, *B* in ${\mathscr {E}}$ is given by

32 $$\begin{aligned} \langle A,B\rangle = \text {Tr}\{A^\dagger B\}. \end{aligned}$$

Clearly, ${\mathscr {E}}$ is a nice error basis. Additionally, we have that if ${\mathscr {E}}_1$ and ${\mathscr {E}}_2$ are nice error bases, then ${\mathscr {E}}^2 = \{\textsf {E}_{1}\otimes \textsf {E}_{2}:E_1\in {\mathscr {E}}_1,E_2\in {\mathscr {E}}_2\}$ is a nice error basis as well. Let ${\mathscr {E}}^N$ be the error basis constructed as *N*-fold tensor product of the Pauli matrices shown in Eq. (31). The error group, denoted by $G_N$, is the vector space over ${\mathbb {C}}$ consisting of the elements in ${\mathscr {E}}^N$.

The symplectic forms introduced in the Results section are based on the commutation relation obtained for the type of errors that we are considering in this paper. In particular, let $A = a_0{\mathbb {I}} + a_1\sigma _x + a_2\sigma _y + a_3\sigma _z$ and $B = b_0{\mathbb {I}} + b_1\sigma _x + b_2\sigma _y + b_3\sigma _z$ be two elements generated by ${\mathscr {E}}$. Then

33 $$\begin{aligned}{}[A,B] = 2i\Big {(}(a_2 b_3 - b_2 a_3)\sigma _x + (a_3 b_1 - b_3 a_1)\sigma _y + (a_1 b_2 - b_1 a_2)\sigma _z\Big {)}. \end{aligned}$$

It follows from the commutation relations of the Pauli operators $[\sigma _i,\sigma _j] = 2i\epsilon _{ijk}\sigma _k$, for $i,j,k = x, y,z$.

#### Proposition 3

(^12^, Theorem 4, Proposition 5, Theorem 6) Let the time evolution be given by the Markovian open system dynamics. Then, the space ${\mathscr {P}} = \text {span}\{\left| \psi _i\right\rangle \}_{i=1,\ldots ,K}$ is a DFS for all time *t* if and only if $J_l\left| \psi _k\right\rangle = c_l\left| \psi _k\right\rangle$, for all $l=1,\ldots ,M$ and $k = 1,\ldots , K$, and the commutator $[H_{ev},J_l]$ has eigenvalues equal to zero for all $\left| \psi _k\right\rangle \in {\mathscr {P}}$, and $l=1,\ldots ,M$. Here

34 $$\begin{aligned} H_{ev} = H_S + \frac{i}{2}\sum _{l = 1}^M \lambda _l(c_l^* J_l - c_l J_l^\dagger ). \end{aligned}$$

Suppose the evolution of a state $\rho (t)$ is given by the Lindblad master equation with the dissipator part described by operators from the set ${\mathscr {J}} = \{J_l:l=1,\dots , M\}$. Assume the existence of a DFS satisfying the assumptions of Proposition 3. Then we can construct the following stabilizer set:

35 $$\begin{aligned} {\mathscr {S}}_\text {DFS}:= \langle S_1, \ldots , S_{M}:S_l = c_l^{-1} J_l, \text { for }l=1,\ldots ,M,\text { where }J_l\in {\mathscr {J}}\rangle . \end{aligned}$$

Suppose ${\mathscr {Q}}$ is the joint eigenspace with eigenvalue $+1$ for every element in ${\mathscr {S}}_\text {DFS}$; i.e., ${\mathscr {S}}_\text {DFS}$ stabilizes ${\mathscr {Q}}$. If $[S_i,S_j] = 0$, for all $i,j=1,\ldots ,M$, then ${\mathscr {S}}_\text {DFS}$ is an abelian group. Furthermore, if the system Hamiltonian $H_{ev}$ belongs to the centralizer $C_{G_N}({\mathscr {S}}_\text {DFS})$, then we can conclude from Proposition 3 that ${\mathscr {Q}}$ is DFS. Similar arguments can be used for sDFS where the stabilizer group is given by ${\mathscr {S}}_\text {sDFS}$ and the commutativity condition is imposed over $H_{S}$.

To show this result, notice that the claim that ${\mathscr {Q}}$ is a stabilizer code of ${\mathscr {S}}_\text {DFS}$ follows from the fact that ${\mathscr {Q}}$ is the nontrivial maximal $+1$-eigenspace of ${\mathscr {S}}_\text {DFS}$. Secondly, for any $\left| \psi \right\rangle \in {\mathscr {Q}}$ and $S_l\in {\mathscr {S}}_\text {DFS}$ we have

36 $$\begin{aligned} J_l\left| \psi \right\rangle = c_l S_l\left| \psi \right\rangle = c_l\left| \psi \right\rangle . \end{aligned}$$

Since $H_S + \frac{i}{2}\sum _{l = 1}^M \lambda _l(c_l^* J_l - c_l J_l^\dagger )$ belongs to $C_{G_N}({\mathscr {S}}_\text {DFS})$, then the commutator of $H_S + \frac{i}{2}\sum _{l = 1}^M \lambda _l(c_l^* J_l - c_l J_l^\dagger )$ with any element in ${\mathscr {S}}_\text {DFS}$ has eigenvalue equal to zero. Therefore, from Eq. (36) and Proposition 3, we have that ${\mathscr {Q}}$ is also a decoherence-free subspace.

### Decoherence-free stabilizer codes for tensor-product noise

Considering the $+_\zeta$ operation defined in Eq. (12) as the sum operation of the additive codes, we derive some constraint over the coordinates of the elements in these codes.

Let *C* be an $+_\zeta$-additive code. If ${\varvec{v}}_1 = ({\varvec{a}}_0,{\varvec{a}}_1,{\varvec{a}}_2,{\varvec{a}}_3)$ and ${\varvec{v}}_2 = ({\varvec{b}}_0,{\varvec{b}}_1,{\varvec{b}}_2,{\varvec{b}}_3)$ are elements in *C*, then

37a $$\begin{aligned} a_{2j} b_{3j}= & {} a_{3j} b_{2j}, \end{aligned}$$

37b $$\begin{aligned} a_{3j} b_{1j}= & {} a_{1j} b_{3j}, \end{aligned}$$

37c $$\begin{aligned} a_{1j} b_{2j}= & {} a_{2j} b_{1j}, \end{aligned}$$

and the following system of equations must also be satisfied

38a $$\begin{aligned} a_{lj}= & {} 0, \end{aligned}$$

38b $$\begin{aligned} a_{ij}= & {} \pm ia_{kj}, \end{aligned}$$

for pairwise distinct $l,i,k\in \{1,2,3\}$ and each $j=1,\ldots ,N$.

To show this result, observe that the set of conditions presented in Eq. (37) follows by imposing commutativity of $+_\zeta$ in Eq. (12). To derive the conditions in Eq. (38), notice that Eq. (37) can be written as

39a $$\begin{aligned} a_{2j} b_{3j} - a_{3j} b_{2j}= & {} 0, \end{aligned}$$

39b $$\begin{aligned} a_{3j} b_{1j} - a_{1j} b_{3j}= & {} 0, \end{aligned}$$

39c $$\begin{aligned} a_{1j} b_{2j} - a_{2j} b_{1j}= & {} 0, \end{aligned}$$

which has a nontrivial solution if and only if $a_{1j} a_{2j} a_{3j} = 0$. Substituting this condition in Eq. (39) and imposing nontriviality to the solution again, we obtain $a_{ij}^2 = - a_{kj}^2$ and $a_{lj} = 0$ for pairwise distinct $l,i,k\in \{1,2,3\}$. Notice that for each *j*, we have independent conditions.

We have presented some intuitions on how to relate operators and vectors (some constraints on the coordinates of the vectors have been presented). However, we need to develop further tools and properties to derive a stabilizer formalism connecting stabilizer and additive codes. In particular, three points are covered in the following subsection. Firstly, we demonstrate that the map $\langle \cdot , \cdot \rangle _\zeta$ is a symplectic form. Using this fact, we show that the map $\zeta$ is an isomorphism between abelian sets of operators and additive codes. Lastly, we introduce symplectic dual codes and the stabilizer formalism connecting quantum stabilizer codes with $+_\zeta$-additive codes.

### Symplectic form and additive codes

A symplectic form connects the centralizer of a stabilizer group to the dual code of the classical code corresponding to the stabilizer group. Symplectic forms can be defined over vector spaces or groups. In the following we consider a symplectic form over groups. Thus, the dual code obtained is an additive code.

A symplectic form over an additive group ${\mathscr {G}}$ to a field *F* is a function

40 $$\begin{aligned} \begin{aligned} f:{\mathscr {G}}\times {\mathscr {G}}&\rightarrow F\\ (g_1,g_2)&\mapsto f(g_1,g_2), \end{aligned} \end{aligned}$$

such that

41a $$\begin{aligned} f(g_1+g_2,g_3)= & {} f(g_1,g_3) + f(g_2,g_3), \end{aligned}$$

41b $$\begin{aligned} f(g_1,g_2)= & {} -f(g_2,g_1), \end{aligned}$$

41c $$\begin{aligned} f(g_1,g_1)= & {} 0, \end{aligned}$$

for all $$g_1,g_2,g_3\in {\mathscr {G}}$$.

For the operation in Eqs. (13-15) to be a symplectic form, the first point we need to show is that the image of $\zeta$ equipped with a proper additive operation forms an additive group.

We claim that the set ${\mathscr {V}} = \zeta (C_{G_N}({\mathscr {S}}))$, where ${\mathscr {S}}$ is a stabilizer group, equipped with $+_\zeta$ operation from Eq (12) is an additive group. Indeed, let ${\varvec{v}}_A,{\varvec{v}}_B,{\varvec{v}}_C\in {\mathscr {V}}$, then the following axioms are satisfied:

1. ${\mathscr {V}}$ is closed under $+_\zeta$;
2. $${\varvec{v}}_A+_\zeta {\varvec{v}}_B = {\varvec{v}}_B +_\zeta {\varvec{v}}_A$$;
3. $$({\varvec{v}}_A+_\zeta {\varvec{v}}_B)+_\zeta {\varvec{v}}_C = {\varvec{v}}_A+_\zeta ({\varvec{v}}_B+_\zeta {\varvec{v}}_C)$$;
4. there exists an element ${\varvec{v}}_{{\mathbb {I}}}$ such that ${\varvec{v}}_A +_\zeta {\varvec{v}}_{{\mathbb {I}}} = {\varvec{v}}_A$;
5. For each ${\varvec{v}}_A\in {\mathscr {V}}$, there exists an element ${\varvec{v}}_B\in {\mathscr {V}}$ such that ${\varvec{v}}_A +_\zeta {\varvec{v}}_B = {\varvec{v}}_{{\mathbb {I}}} = {\varvec{v}}_B +_\zeta {\varvec{v}}_A$.

The first point is clearly true. For the second point, we have that ${\mathscr {V}}$ is the image of $\zeta$ over $C_{G_N}({\mathscr {S}})$. From Eq. (33), we have

42a $$\begin{aligned} a_{2j} b_{3j} - a_{3j} b_{2j}= & {} 0, \end{aligned}$$

42b $$\begin{aligned} a_{3j} b_{1j} - a_{1j} b_{3j}= & {} 0, \end{aligned}$$

42c $$\begin{aligned} a_{1j} b_{2j} - a_{2j} b_{1j}= & {} 0, \end{aligned}$$

for $j=1,\ldots ,N$, where $a_{lj}$ and $b_{pj}$ are the coordinates of the vectors ${\varvec{v}}_A$ and ${\varvec{v}}_B$, respectively, for $l,p=1,2,3$. Thus, we can see from Eq. (12) that $+_\zeta$ is abelian. For the third point, let ${\varvec{v}}_D = {\varvec{v}}_A +_\zeta {\varvec{v}}_B$ and ${\varvec{v}}_E = {\varvec{v}}_B +_\zeta {\varvec{v}}_C$, where each coordinate is given by

43a $$\begin{aligned} d_{0j}= & {} a_{0j} b_{0j} + a_{1j} b_{1j} + a_{2j} b_{2j} + a_{3j} b_{3j}, \end{aligned}$$

43b $$\begin{aligned} d_{1j}= & {} a_{1j} b_{0j} + a_{0j} b_{1j}, \end{aligned}$$

43c $$\begin{aligned} d_{2j}= & {} a_{2j} b_{0j} + a_{0j} b_{2j}, \end{aligned}$$

43d $$\begin{aligned} d_{3j}= & {} a_{3j} b_{0j} + a_{0j} b_{3j}, \end{aligned}$$

and

44a $$\begin{aligned} e_{0j}= & {} b_{0j} c_{0j} + b_{1j} c_{1j} + b_{2j} c_{2j} + b_{3j} c_{3j}, \end{aligned}$$

44b $$\begin{aligned} e_{1j}= & {} b_{1j} c_{0j} + b_{0j} c_{1j}, \end{aligned}$$

44c $$\begin{aligned} e_{2j}= & {} b_{2j} c_{0j} + b_{0j} c_{2j}, \end{aligned}$$

44d $$\begin{aligned} e_{3j}= & {} b_{3j} c_{0j} + b_{0j} c_{3j}, \end{aligned}$$

for $j=1,\ldots ,N$. Then, the result of the sum ${\varvec{v}}_F = {\varvec{v}}_D + {\varvec{v}}_C$ can be described by

45a $$\begin{aligned} f_{0j}= & {} (a_{0j} b_{0j} + a_{1j} b_{1j} + a_{2j} b_{2j} + a_{3j} b_{3j}) c_{0j} + (a_{1j} b_{0j} + a_{0j} b_{1j}) c_{1j}\nonumber \\&+(a_{2j} b_{0j} + a_{0j} b_{2j}) c_{2j} + (a_{3j} b_{0j} + a_{0j} b_{3j}) c_{3j}, \end{aligned}$$

45b $$\begin{aligned} f_{1j}= & {} (a_{1j} b_{0j} + a_{0j} b_{1j}) c_{0j} + (a_{0j} b_{0j} + a_{1j} b_{1j} + a_{2j} b_{2j} + a_{3j} b_{3j}) c_{1j}, \end{aligned}$$

45c $$\begin{aligned} f_{2j}= & {} (a_{2j} b_{0j} + a_{0j} b_{2j}) c_{0j} + (a_{0j} b_{0j} + a_{1j} b_{1j} + a_{2j} b_{2j} + a_{3j} b_{3j}) c_{2j}, \end{aligned}$$

45d $$\begin{aligned} f_{3j}= & {} (a_{3j} b_{0j} + a_{0j} b_{3j}) c_{0j} + (a_{0j} b_{0j} + a_{1j} b_{1j} + a_{2j} b_{2j} + a_{3j} b_{3j}) c_{3j}. \end{aligned}$$

Similarly, it follows that the sum ${\varvec{v}}_{F'} = {\varvec{v}}_A + {\varvec{v}}_E$ is equal to

46a $$\begin{aligned} f'_{0j}= & {} a_{0j} (b_{0j} c_{0j} + b_{1j} c_{1j} + b_{2j} c_{2j} + b_{3j} c_{3j}) + a_{1j} (b_{1j} c_{0j} + b_{0j} c_{1j})\nonumber \\&+a_{2j} (b_{2j} c_{0j} + b_{0j} c_{2j}) + a_{3j} (b_{3j} c_{0j} + b_{0j} c_{3j}), \end{aligned}$$

46b $$\begin{aligned} f'_{1j}= & {} a_{1j} (b_{0j} c_{0j} + b_{1j} c_{1j} + b_{2j} c_{2j} + b_{3j} c_{3j}) + a_{0j} (b_{1j} c_{0j} + b_{0j} c_{1j}), \end{aligned}$$

46c $$\begin{aligned} f'_{2j}= & {} a_{2j} (b_{0j} c_{0j} + b_{1j} c_{1j} + b_{2j} c_{2j} + b_{3j} c_{3j}) + a_{0j} (b_{2j} c_{0j} + b_{0j} c_{2j}), \end{aligned}$$

46d $$\begin{aligned} f'_{3j}= & {} a_{3j} (b_{0j} c_{0j} + b_{1j} c_{1j} + b_{2j} c_{2j} + b_{3j} c_{3j}) + a_{0j} (b_{3j} c_{0j} + b_{0j} c_{3j}). \end{aligned}$$

Rearranging the terms in Eq. (46) and utilizing the relation from Eq. (42), we see that $f_{ij} = f'_{ij}$ for $i=0,1,2,3$ and $j=1,\ldots , N$. Therefore, we have proven Property 3. From the definition of $+_\zeta$ and the relation from Eq. (42), we have that the identity element exists. In particular, the identity element is given by ${\varvec{v}}_{{\mathbb {I}}} = ({\varvec{1}}_{N},{\varvec{0}}_{N},{\varvec{0}}_{N},{\varvec{0}}_{N})$, where ${\varvec{1}}_{N}$ and ${\varvec{0}}_{N}$ are *N*-dimensional vectors with all coordinates equal to 1 and 0, respectively. The same approach can be used to show Property 5.

Now, we can use the previous algebraic structure to show that the expression given in Eqs. (13-15) is a symplectic form. Let ${\varvec{v}}_A = ({\varvec{x}}_0, {\varvec{a}}_1, {\varvec{a}}_2, {\varvec{a}}_3), {\varvec{v}}_B = ({\varvec{x}}_0, {\varvec{b}}_1, {\varvec{b}}_2, {\varvec{b}}_3),$ and ${\varvec{v}}_C = ({\varvec{x}}_0, {\varvec{c}}_1, {\varvec{c}}_2, {\varvec{c}}_3)\in {\mathscr {V}}$. From the clear relationship between Eqs. (13-15), we only need to show that one of these functions is a symplectic form. Then,

47 $$\begin{aligned} \langle {\varvec{v}}_A +_\zeta {\varvec{v}}_B, {\varvec{v}}_C\rangle _{\zeta _{(1,j)}}= & {} (a_{2j}x_{0j} + x_{0j}b_{2j}) c_{3j} - (a_{3j}x_{0j} + a_{0j}x_{3j}) c_{2j}\nonumber \\= & {} (a_{2j} c_{3j} - a_{3j}c_{2j})x_{0j} + (b_{2j} c_{3j} - b_{3j} c_{2j}) x_{0j}\nonumber \\= & {} \langle {\varvec{v}}_A, {\varvec{v}}_C\rangle _{\zeta _{(1,j)}} + \langle {\varvec{v}}_B, {\varvec{v}}_C\rangle _{\zeta _{(1,j)}}, \end{aligned}$$

where $j=1,\ldots ,N$ and we have used the fact that ${\varvec{x}}_{0} = (1,1,\ldots ,1)$. It is also possible to see that

48 $$\begin{aligned} \langle {\varvec{v}}_A, {\varvec{v}}_B \rangle _{\zeta _{(1,j)}}= & {} a_{2j} b_{3j} - a_{3j}b_{2j}\nonumber \\= & {} - (a_{3j}b_{2j} - a_{2j} b_{3j})\nonumber \\= & {} - \langle {\varvec{v}}_B, {\varvec{v}}_A \rangle _{\zeta _{(1,j)}}, \end{aligned}$$

and $\langle {\varvec{v}}_A, {\varvec{v}}_A \rangle _{\zeta _{(1,j)}} = 0$. Thus, we have shown that $\langle \cdot , \cdot \rangle _{\zeta _{(1,j)}}$ is, in fact, a symplectic form.

Similar to previous works on stabilizer codes, we are going to derive a connection between stabilizer codes and classical error-correcting codes. This approach enables us to derive algebraic conditions for the construction and existence of decoherence-free stabilizer codes. We can use it to show the nonexistence of decoherence-free stabilizer codes with some specific parameters.

#### Theorem 4

Let ${\mathscr {V}}_{{\mathscr {S}}_\text {DFS}} = \zeta ({\mathscr {S}}_\text {DFS})$ be a basis of the $+_\zeta$-additive code of the form $C = \{{\varvec{c}}\in {\mathbb {C}}^{4N}|{\varvec{c}} = ({\varvec{c}}_0, {\varvec{c}}_1, {\varvec{c}}_2, {\varvec{c}}_3)\text { where }{\varvec{c}}_0 = (1, 1,\ldots ,1)\in {\mathbb {C}}^N\text { and }{\varvec{c}}_1,{\varvec{c}}_2,{\varvec{c}}_3\in {\mathbb {C}}^N\}$. Then, a decoherence-free stabilizer code ${\mathscr {Q}}$ exists if there exists an $+_\zeta$-additive code *C* over ${\mathbb {C}}$ generated by ${\mathscr {V}}_{{\mathscr {S}}_\text {DFS}}$ such that $C\le C^{\perp _\zeta }$ and $\zeta (H_{ev})\in C^{\perp _\zeta }$.

Indeed, since $C\le C^{\perp _\zeta }$, then for all $S_1,S_2\in {\mathscr {S}}_\text {DFS}$ we have $[S_1,S_2] = 0$. This implies the existence of a maximum joint eigenspace of all operators in ${\mathscr {S}}_\text {DFS}$. Let us denote it by ${\mathscr {Q}}$. In particular, ${\mathscr {Q}}$ is a stabilizer code with the stabilizer given by ${\mathscr {S}}_\text {DFS}$. On the other hand, the hypothesis $\zeta (H_{ev})\in C^{\perp _\zeta }$ leads to $[H_{ev},S_i]=0$ for any $S_i\in {\mathscr {S}}_\text {DFS}$. Therefore, the eigenvalue of the commutator of $H_{ev}$ with any operator in ${\mathscr {S}}_\text {DFS}$ is equal to zero. Using Proposition 3, we have that ${\mathscr {Q}}$ is also a decoherence-free subspace.

### Decoherence-free stabilizer codes for general noise

Let $A,B\in {\mathscr {L}}({\mathbb {C}}^{2N})$ be operators. We define the sum of the vectors $\text {vec}(A)$ and $\text {vec}(B)$ by

49 $$\begin{aligned} \text {vec}(A) +_{\text {vec}} \text {vec}(B) = (A\otimes {\mathbb {I}})\text {vec}(B). \end{aligned}$$

If *A* commutes with *B*, then it is clear that $\text {vec}(A) +_{\text {vec}} \text {vec}(B) = \text {vec}(B) +_{\text {vec}} \text {vec}(A)$. Note that $+_{\text {vec}}$ is not the traditional sum of vectors, which always commutes.

We utilize this relationship to show that the result from the previous subsection and the standard stabilizer formalism can be derived from the formulation presented below. Furthermore, the vectorization of the commutator between two operators is used later to construct the symplectic form and the dual code of the additive code.

Let ${\mathscr {S}}$ be a stabilizer set with operators satisfying the structure of the previous subsection. Assume that ${\mathscr {C}}_\zeta = \zeta ({\mathscr {S}})$ and ${\mathscr {C}}_\text {vec} = \text {vec}({\mathscr {S}})$, where the composition of operators in ${\mathscr {S}}$ corresponds to the respective operation of the additive group. Then ${\mathscr {C}}_\zeta \equiv {\mathscr {C}}_\text {vec}$.

In fact, consider a quantum system with $N = 1$. An operator *E* can be written as $E = e_{01}{\mathbb {I}} + e_{11}\sigma _x + e_{21}\sigma _y + e_{31}\sigma _z$ or $E = e_{00}^1\left| 0\right\rangle \left\langle 0\right| + e_{01}^1\left| 0\right\rangle \left\langle 1\right| + e_{10}^1\left| 1\right\rangle \left\langle 0\right| + e_{11}^1\left| 1\right\rangle \left\langle 1\right|$, where $e_{i1}$, $i=0,1,2,3$, and $e_{pq}^1$, $p,q=0,1$, satisfy the relations

50a $$\begin{aligned} e_{00}^1= & {} e_{01} + e_{31}, \end{aligned}$$

50b $$\begin{aligned} e_{01}^1= & {} e_{11} - ie_{21}, \end{aligned}$$

50c $$\begin{aligned} e_{10}^1= & {} e_{11} + ie_{21}, \end{aligned}$$

50d $$\begin{aligned} e_{11}^1= & {} e_{01} - e_{31}, \end{aligned}$$

and

51a $$\begin{aligned} e_{01}= & {} (e_{00}^1 + e_{11}^1)/2, \end{aligned}$$

51b $$\begin{aligned} e_{11}= & {} (e_{01}^1 + e_{10}^1)/2, \end{aligned}$$

51c $$\begin{aligned} e_{21}= & {} (e_{10}^1 - e_{01}^1)/2i, \end{aligned}$$

51d $$\begin{aligned} e_{31}= & {} (e_{00}^1 - e_{11}^1)/2. \end{aligned}$$

Extending these relations to any positive integer *N*, taking into account that the relations are independent from one to another qubit, we obtain

52a $$\begin{aligned} e_{00}^l= & {} e_{0l} + e_{3l}, \end{aligned}$$

52b $$\begin{aligned} e_{01}^l= & {} e_{1l} - ie_{2l}, \end{aligned}$$

52c $$\begin{aligned} e_{10}^l= & {} e_{1l} + ie_{2l}, \end{aligned}$$

52d $$\begin{aligned} e_{11}^l= & {} e_{0l} - e_{3l}, \end{aligned}$$

and

53a $$\begin{aligned} e_{0l}= & {} (e_{00}^l + e_{11}^l)/2, \end{aligned}$$

53b $$\begin{aligned} e_{1l}= & {} (e_{01}^l + e_{10}^l)/2, \end{aligned}$$

53c $$\begin{aligned} e_{2l}= & {} (e_{10}^l - e_{01}^l)/2i, \end{aligned}$$

53d $$\begin{aligned} e_{3l}= & {} (e_{00}^l - e_{11}^l)/2. \end{aligned}$$

for $l=1,\ldots , N$.Thus, it is clear that one can describe a vector in the $\text {vec}$ formulation in terms of the coordinates of the vector in the $\zeta$ formulation. In order to show that these two formulations are equivalent, we need to show that the additive operation in one formulation can be described by the vectors in the other formulation. Let

54 $$\begin{aligned} A= & {} \sum _{i_1,j_1}\cdots \sum _{i_N,j_N} a^1_{i_1 j_1}\cdots a^N_{i_N j_N}\left| i_1\right\rangle \left\langle j_1\right| \cdots \left| i_N\right\rangle \left\langle j_N\right| , \end{aligned}$$

55 $$\begin{aligned} B= & {} \sum _{p_1,r_1}\cdots \sum _{p_N,r_N} b^1_{p_1 r_1}\cdots b^N_{p_N r_N}\left| p_1\right\rangle \left\langle r_1\right| \cdots \left| p_N\right\rangle \left\langle r_N\right| . \end{aligned}$$

Then,

56 $$\begin{aligned} \text {vec}(AB)= & {} \sum _{p_1,r_1}\cdots \sum _{p_N,r_N} b^1_{p_1 r_1}\cdots b^N_{p_N r_N} (A\left| p_1\right\rangle \cdots \left| p_N\right\rangle )\left| r_1\right\rangle \cdots \left| r_N\right\rangle \nonumber \\= & {} \sum _{i_1,\ldots , i_N} \sum _{r_1,\ldots ,r_N} \Big {(}\sum _{p_1,\ldots ,p_N}a^1_{i_1 p_1}b^1_{p_1 r_1}\cdots a^N_{i_N p_N}b^N_{p_N r_N}\Big {)}\left| i_1\right\rangle \cdots \left| i_N\right\rangle )\left| r_1\right\rangle \cdots \left| r_N\right\rangle \nonumber \\= & {} \sum _{i_1,\ldots , i_N} \sum _{r_1,\ldots ,r_N} \Big {[}(\sum _{p_1}a^1_{i_1 p_1}b^1_{p_1 r_1})\cdots (\sum _{p_N}a^N_{i_N p_N}b^N_{p_N r_N})\Big {]}\left| i_1\right\rangle \cdots \left| i_N\right\rangle )\left| r_1\right\rangle \cdots \left| r_N\right\rangle . \end{aligned}$$

We can describe each coordinate by

57 $$\begin{aligned} \text {vec}(AB)_{i_1,\ldots ,i_N,r_1,\ldots ,r_N} = (\sum _{p_1}a^1_{i_1 p_1}b^1_{p_1 r_1})\cdots (\sum _{p_N}a^N_{i_N p_N}b^N_{p_N r_N}). \end{aligned}$$

From Eq. (52), denoting $\lambda _{i_2,\ldots ,i_N,r_2,\ldots ,r_N} = (\sum _{p_2}a^2_{i_2 p_2}b^2_{p_2 r_2})\cdots (\sum _{p_N}a^N_{i_N p_N}b^N_{p_N r_N})$, we obtain

58a $$\begin{aligned} \text {vec}(AB)_{0,i_2,\ldots , i_N,0,r_2,\ldots ,r_N}= & {} [(a_{01} + a_{31})(b_{01} + b_{31}) + (a_{11} - ia_{21})(b_{11} + ib_{21})]\lambda _{i_2,\ldots ,i_N,r_2,\ldots ,r_N}, \end{aligned}$$

58b $$\begin{aligned} \text {vec}(AB)_{0,i_2,\ldots , i_N,1,r_2,\ldots ,r_N}= & {} [(a_{01} + a_{31})(b_{11} - ib_{21}) + (a_{11} - ia_{21})(b_{01} - b_{31})]\lambda _{i_2,\ldots ,i_N,r_2,\ldots ,r_N}, \end{aligned}$$

58c $$\begin{aligned} \text {vec}(AB)_{1,i_2,\ldots , i_N,0,r_2,\ldots ,r_N}= & {} [(a_{11} + ia_{21})(b_{01} + b_{31}) + (a_{01} - a_{31})(b_{11} + ib_{21})]\lambda _{i_2,\ldots ,i_N,r_2,\ldots ,r_N}, \end{aligned}$$

58d $$\begin{aligned} \text {vec}(AB)_{1,i_2,\ldots , i_N,1,r_2,\ldots ,r_N}= & {} [(a_{11} + ia_{21})(b_{11} - ib_{21}) + (a_{01} - a_{31})(b_{01} - b_{31})]\lambda _{i_2,\ldots ,i_N,r_2,\ldots ,r_N}. \end{aligned}$$

Expanding $\lambda _{i_2,\ldots ,i_N,r_2,\ldots ,r_N}$ in terms of $a_{ij}$ and $b_{ij}$, we see that $\text {vec}(AB)$ can be computed from the vector representation given in Eq. (7). Similarly, Eq. (53) can be applied in order to describe $\zeta (AB)$ in terms of $\text {vec}(A)$ and $\text {vec}(B)$.

Let ${\mathscr {S}}$ be a stabilizer group with operators satisfying the structure of the standard stabilizer formalism. Assume that ${\mathscr {C}}$ is the additive group constructed using the standard stabilizer formalism and ${\mathscr {C}}_\text {vec} = \text {vec}({\mathscr {S}})$, where the composition of operators in ${\mathscr {S}}$ corresponds to the respective operation of the additive group. Then ${\mathscr {C}} \equiv {\mathscr {C}}_\text {vec}$.

To show this result, consider the single qubit $N = 1$ case. Let $A = X(a)Z(b)$, for $a,b\in {\mathbb {Z}}_2$. Then we can write

59 $$\begin{aligned} A = A_{00}\left| 0\right\rangle \left\langle 0\right| + A_{01}\left| 0\right\rangle \left\langle 1\right| + A_{10}\left| 1\right\rangle \left\langle 0\right| + A_{11}\left| 1\right\rangle \left\langle 1\right| , \end{aligned}$$

where $A_{00} = 1 - a$, $A_{01} = (-1)^b a$, $A_{10} = a$, $A_{11} = (-1)^b(1 - a)$. These equalities are clearly invertible. Now, consider the case where $N>1$. The coordinates of $\text {vec}(AB)$ are given by

60 $$\begin{aligned} \text {vec}(AB)_{i_1,\ldots ,i_N,r_1,\ldots ,r_N}= & {} (\sum _{p_1}a^1_{i_1 p_1}b^1_{p_1 r_1})\cdots (\sum _{p_N}a^N_{i_N p_N}b^N_{p_N r_N})\nonumber \\= & {} AB_{i_1 r_1}^1\cdots AB_{i_N r_N}^N. \end{aligned}$$

Then we can see that

61a $$\begin{aligned} AB_{00}^j= & {} (1 - a_1^j)(1 - (-1)^{b_1^j}) + (-1)^{b_1^j} a_2^j a_1^j, \end{aligned}$$

61b $$\begin{aligned} AB_{01}^j= & {} (1 - a_1^j)(-1)^{b_1^j + b_2^j} + (1 - (-1)^{b_1^j})(-1)^{b_2^j} a_2^j a_1^j, \end{aligned}$$

61c $$\begin{aligned} AB_{10}^j= & {} (1 - (-1)^{b_1^j})a_1^j + (-1)^{b_1^j} a_2^j(1 - a_1^j), \end{aligned}$$

61d $$\begin{aligned} AB_{11}^j= & {} a_1^j (-1)^{b_1^j + b_2^j} + (1 - (-1)^{b_1^j})(-1)^{b_2^j}(1 - a_1^j)a_2^j. \end{aligned}$$

Since the above equalities are invertible, we have that both formulations are equivalent.

As explained in the previous section, we need to have a symplectic form to construct the additive code related to the stabilizer code and its centralizer. We can use Eq. (4) to construct the symplectic form used in this subsection.

The map from Eq. (17) is a symplectic form over ${\mathbb {C}}$. Indeed, let $A,B,C\in {\mathscr {L}}({\mathbb {C}}^{2N})$ be operators. First of all, we see that

62 $$\begin{aligned} \langle \text {vec}(A) + \text {vec}(B), \text {vec}(C)\rangle _\text {vec}= & {} \sum _{i=1}^{2N}([(A+B)\otimes {\mathbb {I}} - {\mathbb {I}}\otimes (A+B)^T]\text {vec}(C))_i\nonumber \\= & {} \sum _{i=1}^{2N}[(A\otimes {\mathbb {I}} - {\mathbb {I}}\otimes A^T)\text {vec}(C)]_i + \sum _{i=1}^{2N}[(B\otimes {\mathbb {I}} - {\mathbb {I}}\otimes B^T)\text {vec}(C)]_i\nonumber \\= & {} \langle \text {vec}(A), \text {vec}(C)\rangle _\text {vec} + \langle \text {vec}(B), \text {vec}(C)\rangle _\text {vec}. \end{aligned}$$

The second point follows from

63 $$\begin{aligned} \langle \text {vec}(A), \text {vec}(B)\rangle _\text {vec}= & {} \sum _{i=1}^{2N}[\text {vec}([A,B])]_i\nonumber \\= & {} \sum _{i=1}^{2N}[\text {vec}(AB) - \text {vec}(BA)]_i\nonumber \\= & {} -\sum _{i=1}^{2N}(\text {vec}(BA) - \text {vec}(AB))_i\nonumber \\= & {} -\sum _{i=1}^{2N}[\text {vec}([B,A])]_i\nonumber \\= & {} -\langle \text {vec}(B), \text {vec}(A)\rangle _\text {vec}. \end{aligned}$$

We used the linearity of the vectorization in the second equality. The last point follows by expanding an operator *A* in an eigenbasis and computing $(A\otimes {\mathbb {I}} - {\mathbb {I}}\otimes A^T)\text {vec}(A)$.

Since $\langle \cdot , \cdot \rangle _{\text {vec}}$ gives a symplectic form, Eq. (18) is indeed the dual code of an additive code. Furthermore, we can extend the stabilizer formulation presented in the previous subsection to a larger set of errors.

#### Theorem 5

Let ${\mathscr {V}}_{{\mathscr {S}}_\text {DFS}} = \text {vec}({\mathscr {S}}_\text {DFS})$ be a basis of the $+_\text {vec}$-additive code *C*. Then, a decoherence-free stabilizer code ${\mathscr {Q}}$ exists if there exists an $+_\text {vec}$-additive code *C* over ${\mathbb {C}}$ generated by ${\mathscr {V}}_{{\mathscr {S}}_\text {DFS}}$ such that $C\le C^{\perp _\text {vec}}$ and $\text {vec}(H_{ev})\in C^{\perp _\text {vec}}$.

This result follows the same reasoning used in the previous subsection.

## Data Availability

All data generated or analysed during this study are included in this published article.
